# Recent developments and future avenues for human corticospinal neuroimaging

**DOI:** 10.3389/fnhum.2024.1339881

**Published:** 2024-01-25

**Authors:** Merve Kaptan, Dario Pfyffer, Christiane G. Konstantopoulos, Christine S.W. Law, Kenneth A. Weber II, Gary H. Glover, Sean Mackey

**Affiliations:** ^1^Division of Pain Medicine, Department of Anesthesiology, Perioperative and Pain Medicine, Stanford University School of Medicine, Palo Alto, CA, United States; ^2^Radiological Sciences Laboratory, Department of Radiology, Stanford University School of Medicine, Palo Alto, CA, United States

**Keywords:** corticospinal imaging, corticospinal fMRI, combined brain and spinal cord fMRI, fMRI data analysis, resting-state fMRI, pain processing, motor tasks

## Abstract

Non-invasive neuroimaging serves as a valuable tool for investigating the mechanisms within the central nervous system (CNS) related to somatosensory and motor processing, emotions, memory, cognition, and other functions. Despite the extensive use of brain imaging, spinal cord imaging has received relatively less attention, regardless of its potential to study peripheral communications with the brain and the descending corticospinal systems. To comprehensively understand the neural mechanisms underlying human sensory and motor functions, particularly in pathological conditions, simultaneous examination of neuronal activity in both the brain and spinal cord becomes imperative. Although technically demanding in terms of data acquisition and analysis, a growing but limited number of studies have successfully utilized specialized acquisition protocols for corticospinal imaging. These studies have effectively assessed sensorimotor, autonomic, and interneuronal signaling within the spinal cord, revealing interactions with cortical processes in the brain. In this mini-review, we aim to examine the expanding body of literature that employs cutting-edge corticospinal imaging to investigate the flow of sensorimotor information between the brain and spinal cord. Additionally, we will provide a concise overview of recent advancements in functional magnetic resonance imaging (fMRI) techniques. Furthermore, we will discuss potential future perspectives aimed at enhancing our comprehension of large-scale neuronal networks in the CNS and their disruptions in clinical disorders. This collective knowledge will aid in refining combined corticospinal fMRI methodologies, leading to the development of clinically relevant biomarkers for conditions affecting sensorimotor processing in the CNS.

## Introduction

Neuroimaging of the human brain can provide advanced knowledge of supraspinal correlates of cognitive sequences, emotions, processing of sensory stimuli, and planning and execution of movements (Martucci and Mackey, 2016; Navratilova et al., 2016). The spinal cord−the key relay station between the periphery and the brain−is crucially involved in these processes by transmitting and modulating bidirectional neural information. Imaging biomarkers of both the brain and spinal cord have gained increasing attention and importance in recent years, with improving power to more reliably detect physiologic or pathologic processes within the central nervous system (CNS) (Brown et al., 2011; Nash et al., 2013; Wager et al., 2013; Davis et al., 2017; Lopez-Sola et al., 2017; Weber et al., 2018). Prominent examples include musculoskeletal disorders or chronic pain conditions (Martucci et al., 2014; Martucci and Mackey, 2016; Boissoneault et al., 2017; Mackey et al., 2019). Neuroimaging of the CNS can be further used to shed light on neurobiological changes underlying pharmacological treatment or therapeutic intervention. Upon further development, it has the potential to complement the clinical diagnostic workup or to be employed when planning future clinical trials (Cadotte and Fehlings, 2013; Goldstein-Piekarski et al., 2016).

Spinal cord functional magnetic resonance imaging (fMRI) has trailed brain imaging for many decades (Kornelsen and Mackey, 2007), mainly due to technical hurdles during data acquisition and analysis. However, it is on the rise lately (Nash et al., 2013; Kong et al., 2014; Weber et al., 2018). Technological and methodological innovations in spinal cord fMRI that are overcoming challenges arising from the spinal cord’s size and position/environment (its small size, elongated structure, proneness to field inhomogeneities due to demagnetizing effects resulting from the geometry of the neck between head and upper torso, and the presence of tissues with different magnetic susceptibilities and impact of physiological noise) have recently emerged (Stroman et al., 2014). However, a comprehensive understanding of human sensory and motor systems from basic and clinical perspectives requires measurement of corticospinal activity simultenaously because of their tight neural coupling. This allows the investigation of information processing along entire sensorimotor pathways and the exploration of the interaction between spinal and supraspinal networks. Corticospinal fMRI in humans is still under development due to the profound technical challenges associated with it. However, it is currently progressing, given its promising application in health and disease.

In this review, we will highlight recent innovations in corticospinal fMRI by first covering the distinct protocols developed for different scanner systems (e.g., dynamic per-slice shimming) and discussing custom strategies for corticospinal data processing and analysis (e.g., motion, and physiological noise correction). We will then present applications of corticospinal fMRI in research fields that aim to further the mechanistic understanding of sensory and motor information flow. Further, we will expand this by highlighting its potential use in medical disorders investigating impaired sensorimotor processing along the neuroaxis. We will finally discuss the limitations of corticospinal neuroimaging that remain despite recent developments and give an outlook on potential future directions.

## Technical considerations for corticospinal imaging

Corticospinal fMRI holds challenges in both the data acquisition and processing. Challenges with acquisition are largely due to the inhomogeneity of the main magnetic field (Cohen-Adad et al., 2010; Finsterbusch et al., 2013; Islam et al., 2019). Particularly in the spinal cord, the magnetic susceptibility differences between different tissues (such as vertebral and intervertebral disks) and the geometry of the upper torso-neck-head junction results in an inhomogeneous static magnetic field. The neck is accompanied on top and bottom by structures with substantially larger size, the disposition of which causes demagnetizing effects (Osborn, 1945) that result in large magnetic field gradients. This leads to signal dropouts and distortions in commonly employed T2*-weighted gradient-echo echo-planar imaging (GE EPI) acquisitions (Cooke et al., 2004; Cohen-Adad and Wheeler-Kingshott, 2014; Stroman et al., 2014; Cohen-Adad, 2017) (please note that there are also T2-weighted spin echo acquisition techniques for spinal cord fMRI (Stroman et al., 2018; Staud et al., 2021) that are largely immune to field heterogeneity but result in diminished Blood Oxygen Level Dependent (BOLD) sensitivity. For brevity, we will focus on solutions employed for GE EPI acquisitions in this review). These magnetic field inhomogeneities impact the spinal cord more than the brain. Therefore, custom shimming techniques become necessary to increase field homogeneity (Cohen-Adad and Wheeler-Kingshott, 2014). In addition, for corticospinal fMRI measurements, other typical fMRI acquisition parameters such as in-plane resolution, voxel size, field of view (FOV), echo time, and coil elements need to be optimized individually for the brain and the spinal cord to achieve superior image quality and increased signal-to-noise ratio (Finsterbusch et al., 2013; Tinnermann et al., 2021a). Another major challenge to corticospinal fMRI is the impact of physiological noise. Cardiac and respiratory cycles can result in unwanted fluctuations in the signal, due to the spinal cord’s proximity to the lungs and heart (Brooks et al., 2008; Piche et al., 2009; Cohen-Adad and Wheeler-Kingshott, 2014; Eippert et al., 2017a). The above challenges necessitate developing customized acquisition and analysis strategies rather than standard vendor-provided protocols.

**FIGURE 1:**
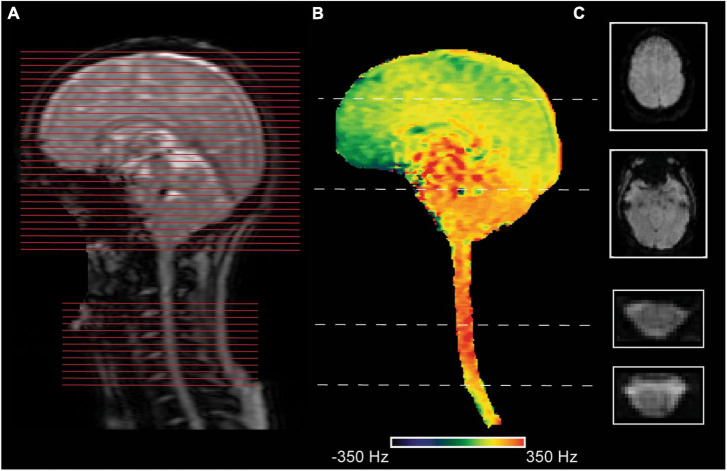
An exemplary corticospinal imaging acquisition for a participant. The acquisition setup was similar to Islam et al. (2019). **(A)** A sagittal localizer image is shown to indicate the position of slices (red lines) for the brain and the spinal cord. The spinal slices were centered in the middle of the cervical vertebra C5. **(B)** A sagittal field map was masked to depict field variations in the tissues of interest. **(C)** Two exemplary slices from the brain (resolution: 3.45 × 3.45 × 5 mm^3^) and the spinal cord (resolution: 1.25 × 1.25 × 5 mm^3^) were acquired with a gradient-echo echo-planar imaging (GE EPI) sequence (TR = 2.5 sec) at a 3T General Electric (GE) Signa system are shown.

Several acquisition techniques have been proposed to tackle these issues. Finsterbusch et al. (2013) first proposed a dynamic shimming method for simultaneous acquisitions by updating linear shims for the brain and cord subvolumes/blocks. Chu et al. (2023) expanded their initial method (Finsterbusch et al., 2013) and developed a dedicated shim algorithm that allows joint optimization of second-order and linear shim terms for each region in a single run in a semi-automated fashion (with minimal interaction by the user). Islam et al. (2019) developed a per-slice shimming approach that dynamically adjusts linear shims (x, y, z, and center frequency) for each brain and spinal cord slice (Figures 1A–C). While brain slices were acquired using a conventional Hamming-weighted since radiofrequency (RF) pulse (22 cm FOV), spinal cord slices were acquired at a reduced FOV (8 cm) using an echo-planar RF pulse. The image quality of their T2* images is comparable to those obtained by the GRASS technique (Frahm et al., 1986; Pui and Fok, 1995; Patronas et al., 2003), which is immune to off-resonance and distortion. They showed robust group activation in the brain and spinal cord from a motor task (Islam et al., 2019).

Spinal cord fMRI also has methodological challenges concerning image processing and analysis. These include the small size of the cord’s gray matter, which is first surrounded by white matter and then pulsatile cerebrospinal fluid (CSF) and vertebral disks. These result in chemical shift effects (Madi et al., 2001) and signal dropout. Notably, several of the procedures that are available in commonly available fMRI analysis software packages, such as FMRIB Software Library (FSL) (Jenkinson et al., 2012), Statistical Parametric Mapping (SPM) (Ashburner, 2012), and Analysis of Functional NeuroImages (AFNI) (Cox, 1996) are tailored for processing of the brain fMRI data. In contrast, spinal cord fMRI data analysis requires special considerations to improve the data quality (Eippert et al., 2017a; Kinany et al., 2022). Thanks to processing and analysis tools like the Spinal Cord Toolbox (SCT) (De Leener et al., 2017) and NEPTUNE toolbox (Deshpande and Barry, 2022), including templates for normalization (De Leener et al., 2018), standard analysis pipelines have started to be established.

In preprocessing, motion correction is a crucial consideration due to the articulated geometry of the spinal cord and the influence of physiological noise. Slice-wise motion correction that allows non-rigid transformations (as implemented in SCT and NEPTUNE toolbox) (Cohen-Adad, 2017; Deshpande and Barry, 2022) may be better suited for spinal cord fMRI than conventional rigid-body motion correction commonly employed for brain imaging. Different motion-correction techniques (slice-wise or rigid-body) have been employed by different spinal and corticospinal fMRI studies (Weber et al., 2016a; Tinnermann et al., 2017; Kinany et al., 2019; Oliva et al., 2022), and further investigation is necessary to compare the efficacy of different motion-correction techniques (Dehghani et al., 2020).

Additionally, physiological noise correction is of utmost importance for spinal cord image analysis. Though many techniques exist (see Eippert et al. (2017a) for a review), the model-based Physiological Noise Modeling (PNM) technique has been commonly employed (Brooks et al., 2008) for spinal fMRI. PNM creates regressors using external physiological (cardiac and respiratory) recordings and utilizes Fourier expansions to model the MRI signal as suggested by Glover et al. (2000). Moreover, the CSF signal is regressed out, as pulsatile flow in CSF will contaminate the signal and hamper the detection of BOLD activity (Eippert et al., 2017a). For a detailed overview of corticospinal imaging acquisition and processing, see Tinnermann et al. (2021a).

## Applications of corticospinal imaging

### Pain processing and modulation

**FIGURE 2:**
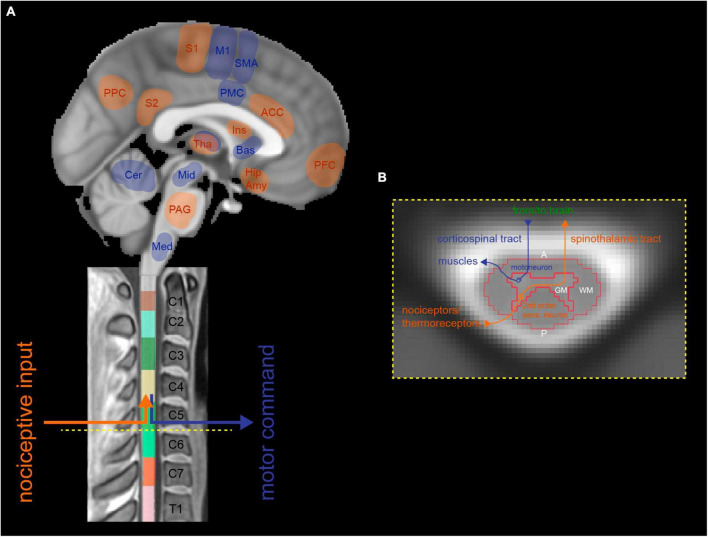
Representative illustration of sensory (i.e., spinothalamic tract) and motor (i.e., corticospinal tract) pathways running along the neuroaxis, connecting distinct supraspinal and cervical spinal neuronal networks. **(A)** Sagittal brain and spinal cord images that show afferent tracts (highlighted in red) transmitting noxious sensory information from the periphery to pain-processing cerebral regions, via second-order sensory neurons in the spinal cord. Efferent tracts (highlighted in blue) relay motor information from related areas in the brain to muscles in the periphery, via motoneurons in the corresponding level of the spinal cord. **(B)** Neurobiological organization of a schematic axial cross-section of the cervical spinal cord. First-order sensory neurons transmitting information from nociceptors/thermoreceptors enter the spinal cord via the dorsal root and connect to second-order sensory neurons in the spinal dorsal horn, which then cross the midline and ascend to the brain as the spinothalamic tract (highlighted in red). Voluntary motor information is sent from the brain’s upper motoneurons via the corticospinal tract through the spinal cord where it is relayed to lower motoneurons located in the spinal ventral horn and further sent to corresponding muscles in the periphery (highlighted in blue). A, anterior; ACC, anterior cingulate cortex; Amy, amygdala; Bas, basal ganglia; Cer, cerebellum; GM, gray matter; Hip, hippocampus; Ins, Insula; M1, primary motor cortex; Med, medulla; Mid, midbrain; P, posterior; PAG, periaqueductal gray; PFC, prefrontal cortex; PMC, premotor cortex; PPC, posterior parietal cortex; S1, primary somatosensory cortex; S2, secondary somatosensory cortex; SMA, supplementary motor area; Tha, Thalamus; WM, white matter.

Based on human neuroimaging of the brain and spinal cord, experimental pain paradigms have identified key processing regions (Martucci and Mackey, 2018). Cerebral regions include primary and secondary somatosensory cortices, insular and cingulate cortices, thalamus, brainstem (e.g., periaqueductal gray (PAG)), and others (Coghill et al., 1999; Fields, 2004; Duerden and Albanese, 2013; Wager et al., 2013) (Figure 2A). They are connected via the ascending pathway to spinal second-order sensory neurons that receive direct input from nociceptors in the periphery. These neurons are primarily located in the dorsal horns of the spinal cord gray matter and can be activated explicitly upon experimental noxious stimulation (Figure 2B). The spinal level and laterality of activation was shown to correspond to the stimulated body area (i.e., dermatome) and body side (Eippert et al., 2009; Sprenger et al., 2012; Weber et al., 2016a; Tinnermann et al., 2021b; Howard et al., 2023). A study imaging the spinal cord and brain of healthy participants in separate sessions applied thermal stimulation at 49°C to the right hand at cervical dermatome C8 and found noxious heat-induced representative activations in the spinal cord, brainstem, and cortical regions that correlated with subjective pain ratings (Khan and Stroman, 2015). These findings suggest a dependency of inter-individual differences in pain processing on brainstem-spinal cord connectivity. However, direct evidence is missing due to the lack of simultaneous spinal cord-brain assessment.

Overcoming technical challenges of corticospinal imaging allows us to explore bottom-up nociceptive processing and top-down pain-modulatory pathways via the brainstem and the intricate interplay between spinal and supraspinal networks during pain perception. Corticospinal fMRI enables investigating temporal associations of activated regions along the CNS. Increased experimental pain-induced activity in the spinal dorsal horn (dermatome C6) and brainstem areas could be reproduced using the combined imaging approach in healthy participants (Ghazni et al., 2010; Cahill and Stroman, 2011; Bosma et al., 2015; Rempe et al., 2015; Sprenger et al., 2018; Stroman et al., 2018; Koning et al., 2023). Furthermore, it could be extended to observations of pathologically altered activation and spinal cord-brainstem connectivity related to pain modulation in spinal cord injury (Stroman et al., 2016), fibromyalgia (Ioachim et al., 2022), and women with provoked vestibulodynia (Yessick et al., 2021). Interestingly, the degree of interaction between spinal circuitries (at the stimulation level) and the PAG (as measured by functional connectivity) during painful heat stimulation was associated with pain intensity ratings of healthy volunteers (Sprenger et al., 2015; Koning et al., 2023). The descending pain-inhibitory connection between the brainstem and spinal networks was further reportedly changed during cognitive/emotional regulation (i.e., inhibition) (Stroman et al., 2011; Oliva et al., 2022) and secondary hyperalgesia (i.e., disinhibition) (Rempe et al., 2014) in healthy participants. Another study found the PAG-spinal cord coupling strength to be related to individual degrees of nocebo effects, highlighting the value of corticospinal imaging to study the descending pain-modulatory system (Tinnermann et al., 2017). Crucially, while all of these studies highlight the spinal-brainstem coupling, only a few used scan protocols that acquired cerebral images beyond the brainstem (Sprenger et al., 2015, 2018; Tinnermann et al., 2017; Oliva et al., 2022). This leaves room for interpretation of higher cortical regions connected to the spinal cord and involved in descending pain modulation. For more reviews on spinal and corticospinal imaging of pain, see Tinnermann et al. (2021a), Kinany et al. (2022), and Haynes et al. (2023).

### Motor task activity

Prominent fMRI brain correlates of motor action and learning, almost exclusively reported for upper limbs in the literature, include primary motor cortex (MI), secondary motor cortices (premotor cortex, supplementary motor area), basal ganglia, and the cerebellum (Dayan and Cohen, 2011; Hardwick et al., 2018; Figure 2A). A limited number of studies have conducted spinal fMRI during motor paradigms since the first one almost three decades ago (Yoshizawa et al., 1996). Task-related activation was consistently found primarily in the spinal ventral horn−with parts of the dorsal horn being co-activated during motor tasks (e.g., task-related sensory feedback) (Kinany et al., 2019)−and ipsilateral to the side of movement, in accordance with the anatomical location of motoneurons within the spinal cord (Weber et al., 2016b; Figure 2B). Depending on the muscles of the upper limbs being activated during the task and accordingly innervated by specific spinal nerves (i.e., myotomes), the rostrocaudal distribution of elicited activation varies along the cervical spinal cord (Stroman et al., 1999; Madi et al., 2001; Maieron et al., 2007; Smith and Kornelsen, 2011; Kinany et al., 2019; Barry et al., 2021). Most recently, a study exploring hand-grasp (i.e., fist-clenching) motor task activity provided more detailed information on the functional organization of the spinal cord (Hemmerling et al., 2023), as compared to previous studies reporting varying localization of rostrocaudal activation (Stroman et al., 1999; Stroman and Ryner, 2001; Giulietti et al., 2008; Ng et al., 2008; Islam et al., 2019). Spinal fMRI revealed areas of activity spanning cervical levels C5-C8, primarily focusing on the C7 spinal cord segment (Hemmerling et al., 2023).

Few studies have deployed corticospinal fMRI during motor tasks, and all were conducted in healthy participants. Two of them used a unilateral hand-grasping task (Islam et al., 2019; Braass et al., 2023). They reported predominantly ipsilateral activation in spinal ventral horns comprising levels C6-C8 and contralateral supraspinal primary and secondary motor areas. This confirms previous findings in the spinal cord and brain imaged separately and with neuroanatomical motor pathways. Importantly, imaging data in these studies were acquired on different scanners, highlighting the reproducibility and generalizability of the findings. In another study, subjects learned motor sequences by performing finger-tapping tasks of different complexities (Vahdat et al., 2015). Distinct spinal learning-related activity modulation was shown at levels C6-C8 as a function of task complexity and independent of changes in related supraspinal regions. The spinal cord’s intrinsic plasticity was characterized by a change in supraspinal-spinal functional connectivity during motor skill acquisition, with the initial relationship between sensorimotor areas and the spinal cord decreasing over time and the latter becoming more independent. This was expanded by another study (Khatibi et al., 2022) in which participants executed a motor sequence learning task (i.e., wrist-controlled joystick movements) for a longer period to examine whether spinal networks and brain-spinal cord circuits are similarly engaged as during the early phase in the previous study. Over time, spinal activation shifted rostrally from C8 to C6-C7 and a change in functional connectivity from sensorimotor to parietal and cerebellar regions was observed, reflecting the acquired finer and automated control of wrist muscles. Recently, the findings of this study were replicated using an innovative data-driven multivariate approach (Kinany et al., 2023) which shows great promise to probe CNS signatures of human sensorimotor behavior in health and disease. For more reviews on spinal and corticospinal imaging of motor task activity, see Landelle et al. (2021), Kinany et al. (2022), and Haynes et al. (2023).

### At-rest functional connectivity

Resting-state functional connectivity (rsFC) −the investigation of intrinsic fluctuations of low frequency fMRI signal within the CNS absent of any stimuli or task−holds great potential both from the basic science and clinical perspectives. Regarding the former, it might help delineate the building blocks of the nervous system; for the latter it might serve to identify abnormalities in various disorders (Fox and Raichle, 2007; Lee et al., 2013; Smith et al., 2013). The resting-state networks have been well identified for the brain (Raichle et al., 2001; Greicius et al., 2003; Damoiseaux et al., 2006) and more recently for the spinal cord (Barry et al., 2014; Kong et al., 2014; Eippert et al., 2017b; Wu et al., 2018, 2019; Kinany et al., 2020; Kaptan et al., 2023), suggesting that rsFC might be a property of the whole CNS. However, the underlying neurophysiological functions of spinal cord resting-state networks remain unknown. It is unclear whether they reflect the communication between brain and cord, local processing within the cord, or the incoming input from the periphery (Eippert and Tracey, 2014). Though these options are not mutually exclusive, corticospinal imaging might elucidate functional properties of resting-state networks in the CNS. However, the investigation of rsFC between the cord and brain has been very limited to date.

Harita and Stroman (2017) and Harita et al. (2019) identified functional connectivity between the brainstem and spinal cord regions using combined brainstem-spinal cord fMRI. Interestingly, the connectivity between the brain and spinal cord was better detected using structural equation modeling, which allows the modeling of complex networks with multiple driving inputs. This contrasts with pairwise correlations between brainstem and cord regions. This result supported the conclusion that brainstem-cord networks may be more complex and more challenging to delineate than within-cord and brain/brainstem networks. Vahdat et al. (2015) investigated the resting-state functional connectivity networks spanning the cervical cord and the brain in healthy adults. They have employed region-of-interest-based and data-driven (ICA) functional connectivity approaches. In line with the functional neuroanatomy, they have observed that: (i) each spinal hemicord preferentially connects to the contralateral hemisphere and (ii) dorsal (sensory) and ventral (motor) regions of the spinal cord demonstrated distinct functional connectivity profiles with the somatosensory and motor brain areas, respectively.

## Discussion

Researchers have recently used corticospinal fMRI to explore sensorimotor projections at different hierarchical levels along the neuroaxis including the cervical spinal cord, brainstem, cerebellum, subcortical structures, and cortical regions. A systemic assessment of neural activity during rest, sensory stimulation, or upon execution of a motor task is necessary to get better insights into the flow of information along distinct sensorimotor regions of the neuroaxis and their intricate interplay. Compared to imaging the brain or spinal cord separately, corticospinal fMRI can capture neural activity in relevant areas of the CNS involved in upper limb sensorimotor processing and characterize entire circuitries rather than isolated networks. However, this technique is currently restricted to cervical levels of the spinal cord and does not allow the investigation of neural activity from lower limb stimulations/tasks.

While many studies exist exploring bidirectional pain processing and modulation along the spinal-brainstem circuitry (Ghazni et al., 2010; Cahill and Stroman, 2011; Bosma et al., 2015; Rempe et al., 2015; Stroman et al., 2016, 2018; Sprenger et al., 2018; Yessick et al., 2021; Ioachim et al., 2022; Koning et al., 2023), only a handful included supraspinal cortical regions to explore sensory (Sprenger et al., 2015, 2018; Tinnermann et al., 2017; Oliva et al., 2022) or motor (Vahdat et al., 2015; Islam et al., 2019; Khatibi et al., 2022; Braass et al., 2023) information flow along the neuroaxis. Methodological developments intended to overcome challenges in image acquisition and data processing are recently evolving (Cohen-Adad et al., 2010; Finsterbusch et al., 2013; Islam et al., 2019; Finsterbusch and Chu, 2023). Corticospinal fMRI is important to characterize pathways processing and modulating pain, motor action, and learning. Recent studies highlight the crucial value of neuroimaging-based biomarkers, such as aberrant activity along sensory/motor circuits, for improved diagnosis, outcome prediction, and tracking of disease (Horn et al., 2016; Martucci and Mackey, 2018; Freund et al., 2019; Mackey et al., 2019; Dahlqvist et al., 2020; Davis et al., 2020; Pfyffer et al., 2020, 2021). Identifying such promising imaging biomarkers could benefit clinical practice by identifying residual neuronal function or spinal plasticity in conditions affecting sensory or motor processing and leading to more personalized interventions (Cadotte and Fehlings, 2013; Goldstein-Piekarski et al., 2016). A potential future application of corticospinal imaging could be to understand the mechanisms of neuromodulation for pain relief or motor recovery. For instance, repetitive transcranial magnetic stimulation (rTMS) is a non-invasive and non-pharmacological neuromodulation technique that is also employed for pain treatment (Lefaucheur et al., 2020; Yu et al., 2020). It is suggested that rTMS applied over the motor cortex (contralateral to the pain site) inhibits the activity in brain regions associated with pain processing such as the anterior cingulate cortex which then triggers descending inhibitory pathways that act at the level of the spinal cord (Leo and Latif, 2007; Leung et al., 2009; Lefaucheur et al., 2020; Yang and Chang, 2020). However, the neuronal mechanisms underlying pain relief are not yet fully understood and most likely involve the interaction of multiple regions and pathways. Corticospinal fMRI through its ability to assess the responses and interactions along the whole neuroaxis could provide a better understanding of neurophysiological processes underlying analgesic mechanisms of rTMS. This may lead to the development of more effective and targeted treatments.

To evaluate corticospinal neuroimaging in the clinical realm, replicability and reproducibility of findings from recent corticospinal fMRI studies must be assessed. Additionally, we firmly believe that open science practices (i.e., data sharing, code sharing) will foster the development of corticospinal fMRI methods and analysis. Crucially, while we have focused on fMRI in this review, there are other non-invasive neuroimaging techniques such as magnetospinography (MSG) (Curio et al., 1991; Sumiya et al., 2017; Sakaki et al., 2020; Akaza et al., 2021; Hashimoto et al., 2022; Mardell et al., 2022) based on super-conducting quantum interference devices (SQUIDs) and non-invasive electrospinography (ESG) (Liberson et al., 1966; Cracco, 1972, 1973; Matthews et al., 1974; Jones, 1977). Similar to corticospinal fMRI, MSG is technically demanding. At the same time, non-invasive ESG recording of somatosensory-evoked potentials via surface electrodes has been employed since the 60s (Namerow, 1968; Small et al., 1978). Despite mainly used for clinical purposes, these technologies should be further explored and evaluated for potential use in basic neuroscientific research investigating corticospinal interactions during sensorimotor tasks (Rocchi et al., 2018; Boehme et al., 2019; Pietro et al., 2021; Chander et al., 2022; Fabbrini et al., 2022; Nierula et al., 2022).

While corticospinal fMRI is in its infancy, it is an up-and-coming tool both for translational neuroscientific discovery and in the clinical setting. It has the potential to further our mechanistic understanding of neurobiological correlates of physiological and pathological information processing and eventually help to develop and evaluate new therapeutic approaches.

## Author contributions

MK: Visualization, Writing−original draft, Writing−review and editing. DP: Visualization, Writing−original draft, Writing−review and editing. CK: Writing−review and editing. CL: Writing−review and editing. KW: Writing−review and editing. GG: Supervision, Writing−review and editing. SM: Supervision, Writing−review and editing.
